# Effect of data binning and frame averaging for micro-CT image acquisition on the morphometric outcome of bone repair assessment

**DOI:** 10.1038/s41598-022-05459-6

**Published:** 2022-01-26

**Authors:** Milena Suemi Irie, Rubens Spin-Neto, Juliana Simeão Borges, Ann Wenzel, Priscilla Barbosa Ferreira Soares

**Affiliations:** 1 Department of Periodontology and Implantology, School of Dentistry, Federal University of Uberlândia, Avenida Pará s/no, Campus Umuarama, Bloco 4L, Bairro Umuarama, Uberlândia, Minas Gerais 38400-902 Brazil; 2 Department of Dentistry and Oral Health, Section for Oral Radiology, Aarhus University, Aarhus C, Denmark

**Keywords:** Data acquisition, Image processing, Preclinical research

## Abstract

Despite the current advances in micro-CT analysis, the influence of some image acquisition parameters on the morphometric assessment outcome have not been fully elucidated. The aim of this study was to determine whether data binning and frame averaging affect the morphometric outcome of bone repair assessment using micro-CT. Four Wistar rats’ tibiae with a surgically created bone defect were imaged with micro-CT six times each, frame averaging set to 1 and 2, and data binning set to 1, 2 and 4, for each of the averaging values. Two-way ANOVA followed by Bonferroni tests assessed the significance of frame averaging and data binning on a set of morphometric parameters assessed in the image volumes (p < 0.01). The effect of frame averaging was not significant for any of the assessed parameters. Increased data binning led to larger trabecular thickness. In contrast, smaller bone volume fraction and bone volume were found as data binning increased. Trabeculae number and trabecular separation were not influenced by any of the parameters. In conclusion, the morphometric outcome of bone repair assessment in micro-CT demonstrated dependency upon data binning, but not frame averaging. Therefore, image acquisition of small anatomical structures (e.g., rat trabeculae) should be performed without data binning.

## Introduction

Bone is a dynamic and highly plastic tissue, and its repair process involves several events to achieve bone formation and to restore possible areas of damage^1,2^. Systemic and local conditions such as osteoporosis^3^, uncontrolled diabetes^4^ and radiotherapy^5^ have been associated with an arrest of physiological bone formation and microarchitecture deterioration^6^. Comprehension of the mechanical and biological behavior of bone tissue as well as the therapeutic strategies in compromised situations is necessary to overcome the impairment caused by these conditions^7^.

Microtomographic (micro-CT) analysis in rodents provides a novel perspective of therapies that aim at bone healing enhancement. Animal models of several diseases, treatments, and the pre-clinical understanding of disease manifestations are commonly assessed using micro-CT. Some authors suggest micro-CT as the reference method to assess bone tissue microarchitecture in three dimensions, and complementing traditional histomorphometry^8–11^. The non-destructive technique, convenience, time save, and the volumetric analyses provided by micro-CT are appealing to analyze cortical and trabecular bone architecture^12–14^.

Due to the polychromatic characteristics of the X-ray beam in micro-CT, several parameters can be set during image acquisition to improve the signal-to-noise ratio (SNR)^15^. SNR is typically defined as the ratio between image signal and image noise and compares the level of the desired signal to the level of the undesirable background noise within an image^16^. Micro-CT systems operate in the “step-and-shoot” mode. For ex vivo studies, the sample holder rotates while the X-ray source and the detector stay still to acquire projection data from different angles. Thus, multiple images can be acquired at each rotation step (i.e., the same projection angle is re-visited multiple times), that will be averaged to compose the final sinogram originating the image projection. This is defined as frame averaging, and it stated to improve the signal-to-noise ration of micro-CT volumes^8^.

Thus, some image quality parameters can be improved by increasing frame averaging, at the cost of longer scan time^8,13,17^.

During image acquisition each pixel of the detector can be counted individually, providing limited signal-to-noise ratios, or combined, as an attempt to increase signal intensity and reduce the noise in a determined area, in a process defined as pixel binning^17^. Pixel binning combines pixel matrices on the detector resulting in a one larger pixel. When data binning is set to 2, one pixel size of the detector becomes 2 × 2 pixels, and when set to 4, the pixel size is 4 × 4 pixels^17^. A larger (i.e., binned) effective sensor pixel increases the signal-to-noise ratio. The detector hardware allows the pixel combination during image acquisition and generates more signal and less noise at the cost of image resolution, which will be reduced^17^. However, better signal-to-noise is desirable in certain situations, especially when scanning strongly attenuating structures (e.g., cortical bone and bone substitute materials). Increasing the image binning reduces the final file size of the images, thus leading to faster image processing^15,17,18^.

Despite the current advances, the influence of some image acquisition parameters on the morphometric assessment outcome have not been fully elucidated. Image acquisition settings that include data binning and frame averaging information are rarely reported in the literature^17^ and little is known regarding its effects on the results of structural quantification of bone. To our knowledge, no study has evaluated the effect of these parameters when assessing the outcome of bone repair using micro-CT. Thus, the aim of this study was to determine whether data binning and frame averaging affect the morphometric outcome of bone repair assessment in micro-CT. The null hypothesis was that the parameters would not influence the outcome of the micro-CT analysis.

## Methods

This study was carried out following the ethical principles for the care and use of laboratory animals and according to the ARRIVE guidelines^19^. The samples were obtained from a previously approved study in The Committee on the Ethics of Animal Experiments of Universidade Federal de Uberlândia (Protocol Number: 076/17, CEUA-UFU), thus it was exempted from additional reviewing. All methods were performed in accordance with the relevant guidelines and regulations. The surgical procedure was performed as previously described^20^. Briefly, four animals were submitted to general anesthesia intraperitoneally and surgical access to the left tibial metaphysis was obtained. A bone defect was performed with a 1.6 mm ⌀ drill (Neodent, Curitiba, Brazil) at 12,000 rpm, under copious irrigation with sterile saline solution of 0.9% sodium chloride. Suturing was performed with 4–0 nylon surgical monofilament. All procedures were performed by a single operator (MSI). After 7 days, the animals were submitted to euthanasia by intraperitoneal overdose of thiopental (150 mg/kg). The tibiae were removed and covered with moist gauze containing sterile saline solution of 0.9% sodium chloride and frozen at − 20 °C until the time of analysis.

Four Wistar rat tibiae with the bone defect were individually scanned six times in a micro-CT unit (μCT 40, Scanco Medical, Bruttisellen, Switzerland). Each sample was wrapped in wet paper and positioned with the long axis perpendicular to the horizontal plane. The following scanning settings were used: 70 kV, 114 µA, 10 µm isotropic pixel size (leading to 10 µm voxel size), 1 mm-thick Al filter, rotation step of 0.5° and 180° rotation. Image acquisition was performed with frame averaging set to 1 and 2, and data binning set to 1, 2, and 4 for each frame setting.

The raw data of the acquisitions were exported to NRecon software (Bruker, Kontich, Belgium). The software allows standard correction for some artefacts (beam hardening, noise, and ring artefacts) in the volumes. The volumes were reconstructed using 30% of beam hardening correction, the value most often suggested in the literature for ex vivo bone assessment with micro-CT^9^. The region of interest (ROI) was manually draw in each section of the volume to delineate the lesion area. A fixed threshold (50—255), was chosen to segment the bone based on the usual 8-bit calibrated gray scale of micro-CT volumes. Bone volume (BV) (mm^3^), tissue volume (TV) (mm^3^), bone volume fraction (BV/TV), trabecular thickness (Tb.Th), trabeculae number (Tb.N), and trabecular separation (Tb.Sp) of the reconstructed volumes were assessed using the software CtAn (Bruker micro-CT, Kontich, Belgium). A single trained operator (PBFS) performed all analyses.

The system of the device used for image acquisition (Scanco Medical, Bruttisellen, Switzerland) was distinct from the software employed for data reconstruction and analysis (Bruker, Kontich, Belgium). Thus, data binning of pixel matrices during image acquisition were not automatically computed in the results obtained from CtAn (Bruker, Kontich, Belgium). This “scale calibration” was performed post-hoc, by multiplying linear measurements for 2 (binning 2 × 2) and 4 (binning 4 × 4), while volumetric measurements were multiplied for 8 (binning 2 × 2) and 64 (binning 4 × 4).

### Statistical analysis

Statistical analysis was performed using Sigma Plot software (version 13.1; Systat Software Inc., San Jose, CA, USA). Data were tested for normal distribution (Shapiro–Wilk's test) and equality of variances (Levene's test). ANOVA two-way was performed to assess the effect of frame averaging and data binning on the morphometric parameters, and Bonferroni post-hoc test compared the mean and standard deviation of the groups. Due to the multiple tests (n = 5) for each parameter, the Bonferroni correction was applied, which adjusted the significance level from 5 to 1%.

## Results

Table 1 and shows the mean and standard deviations of TV considering the diverse variations of frame averaging and data binning. Two-way ANOVA showed no significant effect of frame averaging (p = 0.63) and data binning (p = 0.78). A possible interaction between these parameters (p = 0.80) was not observed.

**Table 1:** Mean and standard deviation of TV values, according to frame averaging (Avg 1 or Avg 2) and data binning (Bin 1, Bin2, and Bin 4).

	TV (mm^3^)	
	Avg 1	Avg 2
Bin 1	309.4 ± 39.1	291.9 ± 39
Bin 2	289.1 ± 33.4	290.8 ± 23
Bin 4	297.1 ± 12.1	294.8 ± 28

Table 2 presents the results for BV. Two-way ANOVA showed a significant effect of data binning (p = 0.01) however no significant effect was observed for frame averaging (p = 0.91) and for the interaction between these parameters (p = 0.95). There was no difference between data binning 1 and 2 (p = 0.76), or between data binning 2 and 4 (p = 0.11). However, data binning of 1 was statistically different (p = 0.009) from data binning of 4, irrespective of the frame averaging.

**Table 2:** Mean and standard deviation of BV values, according to frame averaging (Avg 1 or Avg 2) and data binning (Bin 1, Bin2, and Bin 4).

	BV (mm^3^)	
	Avg 1	Avg 2
Bin 1	50.2 ± 18.6^A^	48.2 ± 17.3^A^
Bin 2	39.9 ± 14.3^A^	42.0 ± 13.4^A^
Bin 4	24.1 ± 7.4^B^	26.0 ± 10.6^B^

Different uppercase letters among the rows in a column indicate significant differences, Bonferroni post-hoc test (p < 0.05).

In Table 3, the results for BV/TV are presented. The influence of frame averaging and data binning on BV/TV was in agreement with the results from BV. Two-way ANOVA showed a significant effect of data binning (p < 0.001), however no significant effect was observed for frame averaging (p = 0.72) and for the interaction between these parameters (p = 0.99). There was no difference between data binning of1 and 2 (p = 0.66), or between 2 and 4 (p = 0.02). However, data binning set to 1 was statistically different (p < 0.001) from 4, irrespective of the frame averaging.

**Table 3:** Mean and standard deviation of BV/TV values, according to frame averaging (Avg 1 or Avg 2) and data binning (Bin 1, Bin2, and Bin 4).

	BV/TV (%)	
	Avg 1	Avg 2
Bin 1	15.9 ± 4.1^A^	16.2 ± 3.7^A^
Bin 2	13.5 ± 3.5^A^	14.2 ± 3.4^A^
Bin 4	8.1 ± 2.2^B^	8.6 ± 2.8^B^

Different uppercase letters among the rows in a column indicate significant differences; Bonferroni post-hoc test (p < 0.05).

The values of Tb.Th are presented in Table 4. The effect of frame averaging was not significant (p = 0.68). However, data binning had an effect on Tb.Th in all levels (p < 0.001), but without interaction with frame averaging (p = 0.86). Larger data binning led to larger Tb.Th.

**Table 4:** Mean and standard deviation of Tb.Th values, according to frame averaging (Avg 1 or Avg 2) and data binning (Bin 1, Bin2, and Bin 4).

	Tb.Th	
	Avg 1	Avg 2
Bin 1	0.13 ± 0.00^A^	0.14 ± 0.01^A^
Bin 2	0.17 ± 0.01^B^	0.18 ± 0.01^B^
Bin 4	0.27 ± 0.02^C^	0.26 ± 0.03^C^

Different uppercase letters among the rows in a column indicate significant differences, Bonferroni post-hoc test (p < 0.05).

Tb.N and Tb.Sp are presented in Table 5 and 6, respectively. Frame averaging did not affect either Tb.N (p = 0.88), or Tb.Sp (p = 0.81). There was no influence of data binning in Tb.N (p = 0.16) and Tb.Sp (p = 0.17). Thus, no interaction was found between these parameters for either Tb.N (p = 0.96) or Tb.Sp (p = 0.74).

**Table 5:** Mean and standard deviation of Tb.N values, according to frame averaging (Avg 1 or Avg 2) and data binning (Bin 1, Bin2, and Bin 4).

	Tb.N	
	Avg 1	Avg 2
Bin 1	1.20 ± 0.3	1.16 ± 0.6
Bin 2	1.63 ± 0.7	1.65 ± 0.6
Bin 4	1.22 ± 0.4	1.33 ± 0.4

**Table 6:** Mean and standard deviation of Tb.Sp values, according to frame averaging (Avg 1 or Avg 2) and data binning (Bin 1, Bin2, and Bin 4).

	Tb.Sp	
	Avg 1	Avg 2
Bin 1	1.18 ± 0.6	1.30 ± 0.6
Bin 2	1.69 ± 0.3	1.66 ± 0.3
Bin 4	2.01 ± 0.1	1.90 ± 0.1

**Figure 1:**
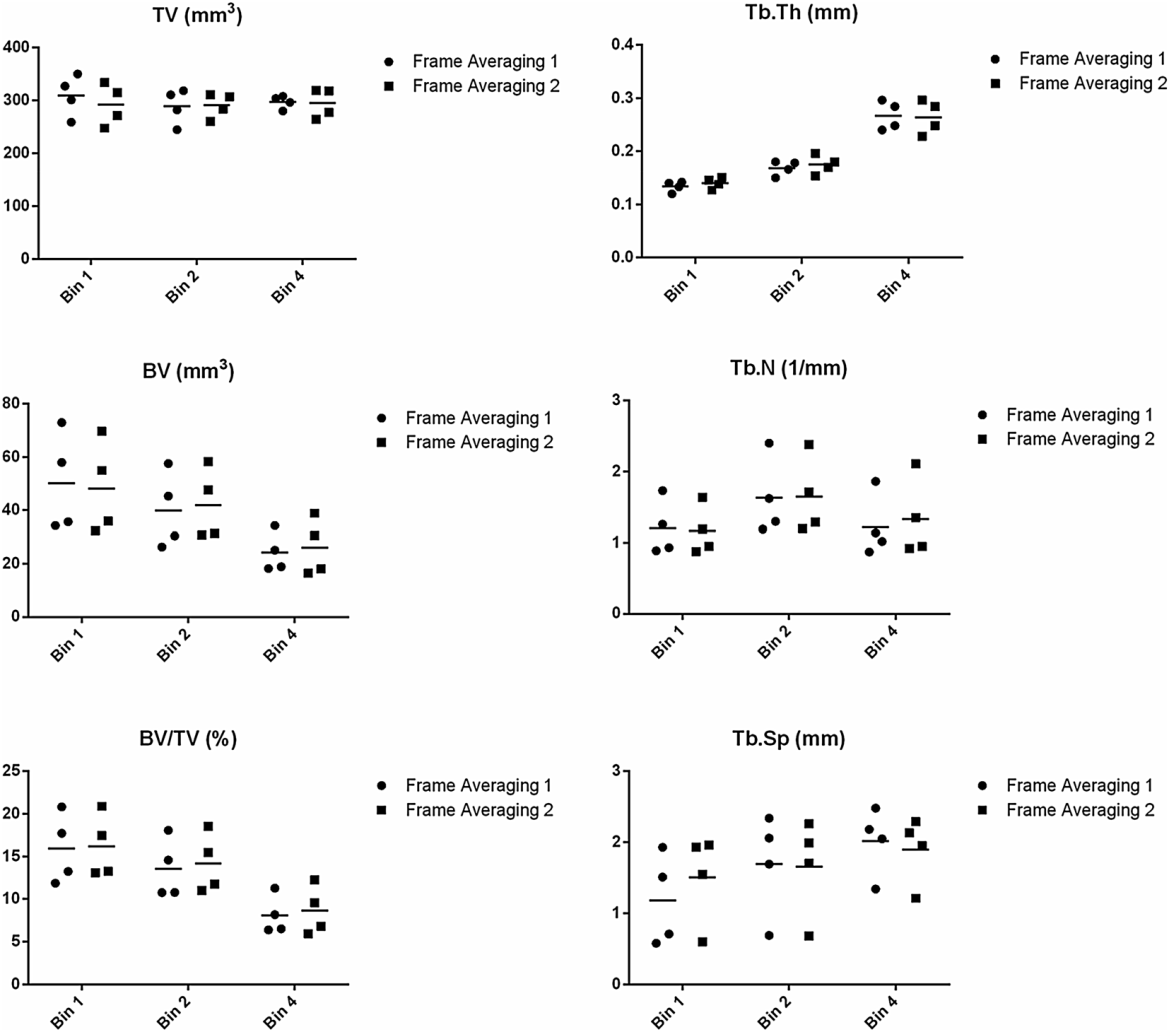
Summary plot for each subject and parameter.

All values that originated the tables are plotted in Fig. 1 allowing a better visualization of the data.

**Figure 2:**
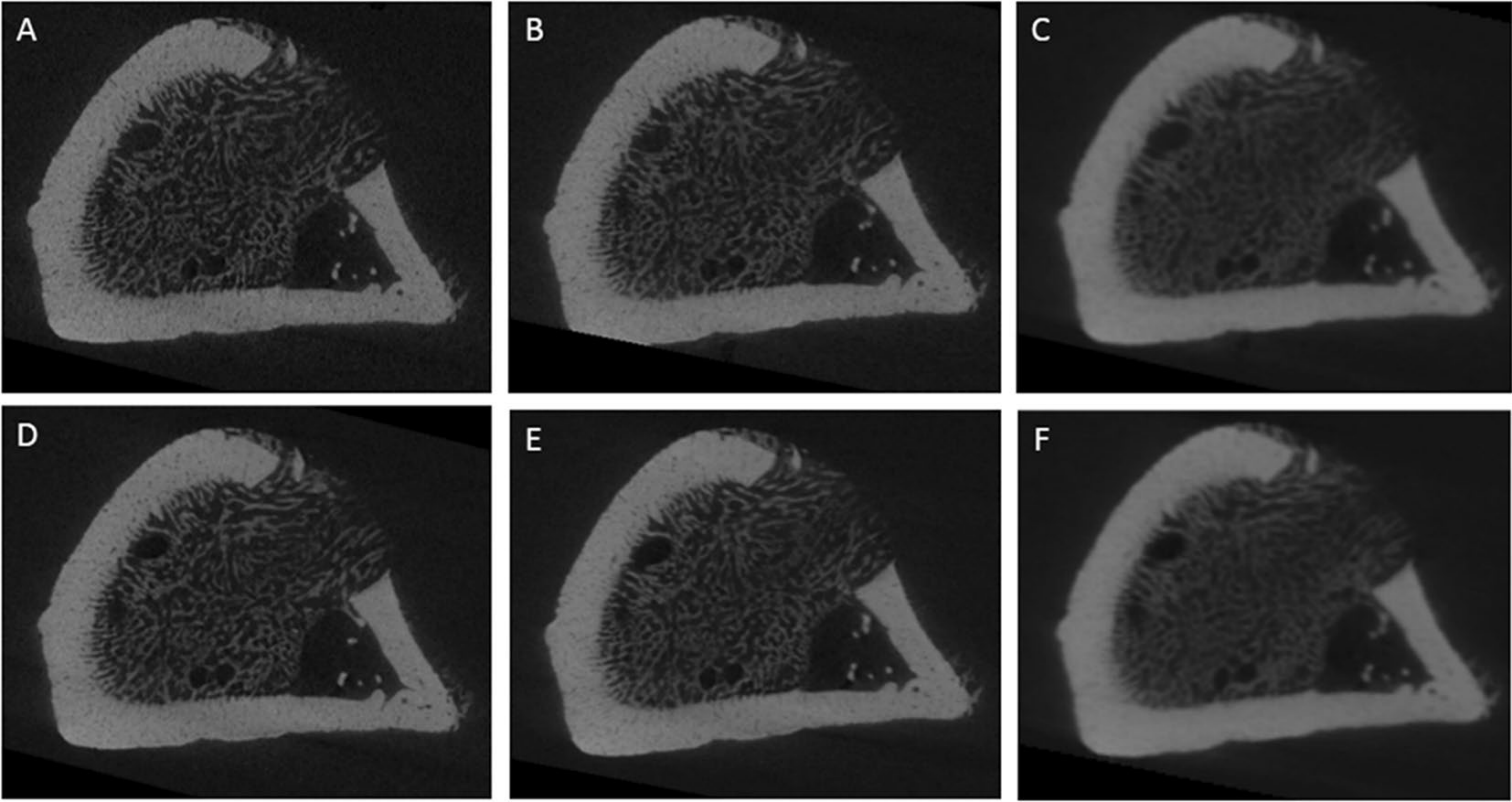
Representative section of the reconstructed data for each tested acquisition parameter: (**A**) Frame averaging = 1, data binning = 1; (**B**) Frame averaging = 1, data binning = 2; (**C**) Frame averaging = 1, data binning = 4; (**D**) Frame averaging = 2, data binning = 2; (**E**) Frame averaging = 2, data binning = 2; (**F**) Frame averaging = 2, data binning = 4.

Figure 2 shows representative sections of the reconstructed data when varying the tested acquisition parameters, focusing on the same region-of-interest in the same sample.

## Discussion

Micro-CT is one of the most used methods to increase knowledge of the microstructure of bone tissues under several pathological conditions. The aim of this study was to determine whether data binning and frame averaging affects the morphometric outcome of bone repair assessment using micro-CT. The results showed that data binning affected some specific bone microstructure measurements. In contrast, no significant difference was observed in the assessed parameters after frame averaging was altered.

The samples of the experiment were sequentially scanned, and the image reconstruction parameters were standardized. All efforts were taken to reduce the influence of confounding factors in the analysis. However, a certain degree of imprecision was unavoidable (i.e., the method has its innate errors). Each sample had its region-of-interest manually delineated because of the different data binning. Thus, minor tissue volume (TV) variations could be detected. Despite that, no significant difference in volume extracted was shown. A drawback of the current methodology is that each sample was scanned only once for each setting (i.e., there was no repetition of the same scanning parameters for the same sample). With this, the exact extension of the method error was not quantified.

Acquiring the images with 10 µm voxel size is considered suitable for the analysis of rat trabeculae^11^, however image quality relies on further factors, including spatial resolution. Spatial resolution is the minimum difference needed between two objects to distinguish them as separate structures in the reconstructed image^21^ and is measured in line-pairs per millimeter^22^. In other words, it indicates the conspicuity to differentiate small structures in the images. The modulation transfer function (MTF) is the objective measurement of spatial resolution through the projection on detectors in X-ray-based tomographic devices. It is a more accurate measure of spatial resolution than visual assessment^23^. The MTF can be influenced by the signal pattern in relation to the detector grid, and the stage of data sampling, including frame averaging and pixel binning^24^. In the present study, binning and frame averaging effect on MTF were not directly assessed. For this purpose, phantoms are generally used^21^. It is important to emphasize, thus, that the voxel size does not equal spatial resolution, as often mistakenly assumed^25^. Despite the fact that the spatial resolution of micro-CT systems is higher than in cone beam computed tomography^25^, its effect on micro-CT analyzes is a relevant topic that should be more often discussed in the literature. This is relevant since the samples scanned in micro-CT are of small dimensions and require high spatial resolution for detecting structural differences and details. (e.g., analyzes of trabecular structures in animals or in studies that assess the degradation of scaffolds in vivo). In addition, image processing can be impacted by other factors rather than voxel size, including image resolution, noise, artefacts, and post-acquisition processing (e.g., filtering)^26^.

Data binning decreases native image resolution. The present findings revealed that data binning affected trabecular thickness measurement significantly. The more the data binning was increased, the larger was Tb.Th. The same behavior was shown by previous studies in which Tb.Th was consistently found to increase as voxel size increased^27,28^. Considering these results, software binning should be avoided, and preference should be given to protocols based on the smaller nominal pixel (and voxel) sizes^29^. This pattern of dependence was not unexpected, since volumes acquired with larger voxel size fail to accurately present small bone trabeculae (partial volume effect)^30^. Even though data binning shows a distinct mechanism that “increases” the pixel/voxel size after signal acquisition, without affecting scanning time^15^, the effect of a volume with a larger voxel size was also noticed in the present study. In the bone defect area, the trabeculae are thinner and closely arranged. Distinct levels of mineralization of the woven bone led to the occurrence of pixels displaying wider gray level ranges in this area, most of them indicating lower attenuation degree. When data binning was used, only the thickest portion of the trabeculae were properly segmented. Thinner and less mineralized structures had their attenuation “dissolved” by pixel binning, following the application of a global threshold to segment the bone, and under influence of the “partial volume” effect. Thus, pixel binning resulted in the underestimation of thin trabeculae and overestimation of thicker trabeculae^30^. Bone volume fraction (BV/TV) and BV reduction also reflects this effect. Although an increase in Tb.N and Tb.Sp values is expected as a result of this trabeculae “disruption” after segmentation, they did not demonstrate dependency on the data binning process. Probably, not only the thinnest portion of the trabeculae was undetected, but also the smallest trabeculae dispersed in the healing area, counterbalancing the measurements.

Signal-to-noise ratio is simultaneously dependent from the number of incident photons in the detector and the sensitivity of the charge-coupled detector^21^. Frame averaging can reduce the noise from intensity fluctuations above and below the actual image intensity^31^. Noise has a random pattern even for constant image acquisition parameters^32^. In the present study the increase in frame averaging (1 to 2) had no effect in the evaluated parameters. This result should be cautiously interpreted. First, only the repair tissue of rat tibia was analyzed. The effect of frame averaging on root resorption volume in rats has been previously demonstrated^17^. Therefore, further studies should investigate if such findings remain when assessing different bony tissues and structures. Besides, Bonferroni correction was applied to reduce inflated type I error since multiple comparisons were performed. An impractical sample size would be required to ensure the frame averaging influence on the analysis of our samples. Considering the ethical aspects of studies in animals, it seemed inappropriate with an enormous increase in the sample size in order to verify the occurrence of small differences in morphometric measurements due to different data averaging values. Moreover, in bone repair investigations, the effects of independent variables are usually great enough to be clinically relevant^33^. This fact would probably make the influence of frame averaging on bone repair analysis irrelevant, if it exists. Despite that, using the same parameter throughout image acquisition of all groups is strongly recommended.

In micro-CT studies two features must be considered: time consumption and data storage. Time consumption is pertinent to both human resources and equipment depreciation. Besides, taking into account in vivo scanning, it is even more critical. It has been demonstrated that a standard micro-CT acquisition resulted in consistently high radiation doses (0.295–0.507 Gy per acquisition)^34^. The radiation levels in micro-CT observed in the mentioned experiment are usually not lethal, but have the potential to influence the experimental outcomes by disturbing the immune response and other physiological processes^35^. Moreover, in vivo micro-CT scanning requires the subject to be anesthetized making longer scanning unfeasible. In this context, increased frame averaging requires longer image acquisition periods^17^. Data binning impacts the size of the volume files^15^. The required disc space for data storage involves some practical questions regarding the maintenance and sharing of large amounts of data (so-called big data)^36^.

Selection of image acquisition parameters during micro-CT analysis may have significant effects on the results and affect the reproducibility of studies^28,37^. There is limited available information regarding standardization of important parameters during acquisition, reconstruction, analysis and reporting of data from bone tissue repair, which makes it difficult to compare among models and study designs. Based on the present results, it is also opportune to study the influence of other image acquisition parameters, and multiple micro-CT devices should be assessed.

Besides the acquisition parameters, the thresholding technique during the analysis can strongly affect the results^38^. There are several segmentation techniques to separate bone from “non-bone” tissue. In this study, a global threshold was applied which is the most widely used technique is built-in image analysis software. It is based on histogram analysis, in which a grayscale value is chosen, and voxels above this value are considered as bone^39^^.^ In the literature, some authors suggest refining this technique can be refined with the application of a voxel-specific local threshold. However, this technique is restricted to analysis of larger structures and samples and would not fit the sample of the present study^38^.

In conclusion, the morphometric outcome of bone repair assessment in micro-CT demonstrated dependency upon data binning. Trabecular thickness was the parameter affected the most. Image acquisition of small structures (e.g., rat trabeculae) should be performed without data binning. In contrast, no difference was observed by increasing the frame averaging value. Further studies should elucidate the influence of different values of averaged projections during micro-CT image acquisition.
